# Chest MRI to diagnose early diaphragmatic weakness in Pompe disease

**DOI:** 10.1186/s13023-020-01627-x

**Published:** 2021-01-07

**Authors:** Laurike Harlaar, Pierluigi Ciet, Gijs van Tulder, Alice Pittaro, Harmke A. van Kooten, Nadine A. M. E. van der Beek, Esther Brusse, Piotr A. Wielopolski, Marleen de Bruijne, Ans T. van der Ploeg, Harm A. W. M. Tiddens, Pieter A. van Doorn

**Affiliations:** 1 Center for Lysosomal and Metabolic Diseases, Department of Neurology, Erasmus MC, University Medical Center Rotterdam, Dr. Molewaterplein 40, 3015 GD Rotterdam, The Netherlands; 2 Departments of Radiology and Nuclear Medicine, Paediatrics, and Respiratory Medicine and Allergology, Erasmus MC, University Medical Center Rotterdam, Rotterdam, The Netherlands; 3 Biomedical Imaging Group Rotterdam, Department of Radiology and Nuclear Medicine, Erasmus MC, University Medical Center Rotterdam, Rotterdam, The Netherlands; 4 Department of Radiology and Nuclear Medicine, Erasmus MC, University Medical Center Rotterdam, Rotterdam, The Netherlands; 5 Department of Computer Science, University of Copenhagen, Copenhagen, Denmark; 6 Center for Lysosomal and Metabolic Diseases, Department of Paediatrics, Erasmus MC, University Medical Center Rotterdam, Rotterdam, The Netherlands

**Keywords:** Pompe disease, Neuromuscular disease, Lysosomal storage disease, MRI, Diaphragm, Respiratory function

## Abstract

**Background:**

In Pompe disease, an inherited metabolic muscle disorder, severe diaphragmatic weakness often occurs. Enzyme replacement treatment is relatively ineffective for respiratory function, possibly because of irreversible damage to the diaphragm early in the disease course. Mildly impaired diaphragmatic function may not be recognized by spirometry, which is commonly used to study respiratory function. In this cross-sectional study, we aimed to identify early signs of diaphragmatic weakness in Pompe patients using chest MRI.

**Methods:**

Pompe patients covering the spectrum of disease severity, and sex and age matched healthy controls were prospectively included and studied using spirometry-controlled sagittal MR images of both mid-hemidiaphragms during forced inspiration. The motions of the diaphragm and thoracic wall were evaluated by measuring thoracic cranial-caudal and anterior–posterior distance ratios between inspiration and expiration. The diaphragm shape was evaluated by measuring the height of the diaphragm curvature. We used multiple linear regression analysis to compare different groups.

**Results:**

We included 22 Pompe patients with decreased spirometry results (forced vital capacity in supine position < 80% predicted); 13 Pompe patients with normal spirometry results (forced vital capacity in supine position ≥ 80% predicted) and 18 healthy controls. The mean cranial-caudal ratio was only 1.32 in patients with decreased spirometry results, 1.60 in patients with normal spirometry results and 1.72 in healthy controls (*p* < 0.001). Anterior–posterior ratios showed no significant differences. The mean height ratios of the diaphragm curvature were 1.41 in patients with decreased spirometry results, 1.08 in patients with normal spirometry results and 0.82 in healthy controls (*p* = 0.001), indicating an increased curvature of the diaphragm during inspiration in Pompe patients.

**Conclusions:**

Even in early-stage Pompe disease, when spirometry results are still within normal range, the motion of the diaphragm is already reduced and the shape is more curved during inspiration. MRI can be used to detect early signs of diaphragmatic weakness in patients with Pompe disease, which might help to select patients for early intervention to prevent possible irreversible damage to the diaphragm.

## Background

Respiratory insufficiency due to muscle weakness is often observed in patients with muscle disease, and is a typical characteristic of patients with advanced Pompe disease, an autosomal recessive metabolic myopathy caused by a deficiency of acid alpha-glucosidase [1–3]. Respiratory dysfunction in Pompe patients is caused mainly by weakness of the diaphragm, and is demonstrated by a decreased forced vital capacity (FVC) particularly in supine position [4, 5]. In adults with non-classic Pompe disease, treatment with enzyme replacement therapy (ERT) resulted in an improved walking distance and muscle strength and stabilizing of respiratory function [6–10]. However, the effect of ERT is much smaller on respiratory function than on skeletal muscle function [7, 9, 10]. The international guideline for starting ERT, indicates that ERT should start when patients have skeletal muscle weakness and/or respiratory muscle weakness defined as an forced vital capacity (FVC) of below 80% predicted [11]. However, it is possible that damage to the diaphragm had already become irreversible before ERT was started. Early recognition of diaphragmatic weakness is important to allow a timely start of treatment, which is even more important as new therapies are currently tested or are in a preclinical phase.

Routine pulmonary function tests, such as vital capacity, FVC and mean inspiratory and expiratory pressures (MIP and MEP) do not differentiate between the function of the diaphragm and the intercostal muscles [5, 12, 13]. Improved insight into the contribution of the different respiratory muscles to inspiration can be provided using spirometry-controlled MRI, evaluating the entire diaphragm and thoracic wall during respiratory movements [14].

Using MRI, the predominantly involvement of the diaphragm in Pompe was shown in patients with advanced muscular involvement [15–18]. However, these studies do not reveal when diaphragmatic weakness starts, and whether an impaired diaphragm function is initially compensated by other respiratory muscles, resulting in normal pulmonary function tests results.

The aim of this study was to identify early signs of diaphragmatic weakness. We therefore used advanced image-analysis techniques to evaluate the motion and shape of the diaphragm in detail. We included a large group of children and adults with Pompe disease covering the spectrum of disease severity, ranging from those with normal spirometry results to those who need nocturnal ventilation due to respiratory dysfunction.

## Methods

### Study design and participants

In this cross-sectional study, we conducted spirometer-controlled MRI scans in Pompe patients (age ≥ 8 years) and healthy controls who had been matched for sex and age. Pompe patients who visited the Center for Lysosomal and Metabolic Diseases at Erasmus MC University Medical Center, the reference center for Pompe disease in the Netherlands, were consecutively invited to participate between January 2016 and February 2018. Inclusion criteria were a confirmed diagnosis of non-classic Pompe disease (based on two disease causing variants in the acid alpha-glucosidase gene and/or decreased enzyme activity in fibroblasts) and the ability to lie in a supine position for at least 30 min without mechanical ventilation. Exclusion criteria were comorbidities or devices that did not permit MRI investigations, and claustrophobia. The protocol was approved by the Medical Ethical Committee at our hospital (MEC-2007-103, amendment 7). All participants provided written informed consent.

### Pulmonary function tests

Before MRI, pulmonary function tests were performed according to ATS/ERS standards [19]. FVC was measured with the patient in upright seated and supine positions. MIP and MEP were measured with the patient in the upright position. All respiration maneuvers were repeated three times and the best effort was used in further analysis. Results were expressed as a percentage of predicted normal values [20, 21].

### MRI analysis

All patients were examined on a 3 T GE Signa 750 MRI (General Electric Healthcare, Milwaukee, Winconsin, USA), using the whole-body coil for radio-frequency excitation and a 32-channel torso coil for signal reception. We developed a new MRI scanning protocol including end-expiration and end-inspiration breath-hold acquisitions, followed by dynamic acquisitions during forced expiration, forced inspiration, and the sniff maneuver (Table 1). To ensure that all breathing maneuvers were executed correctly and with maximum effort, all breathing maneuvers were performed before and during MRI using a MR compatible spirometer, with instructions given through headphone. The overall acquisition time per patient was 20–25 min.

**Table 1:**
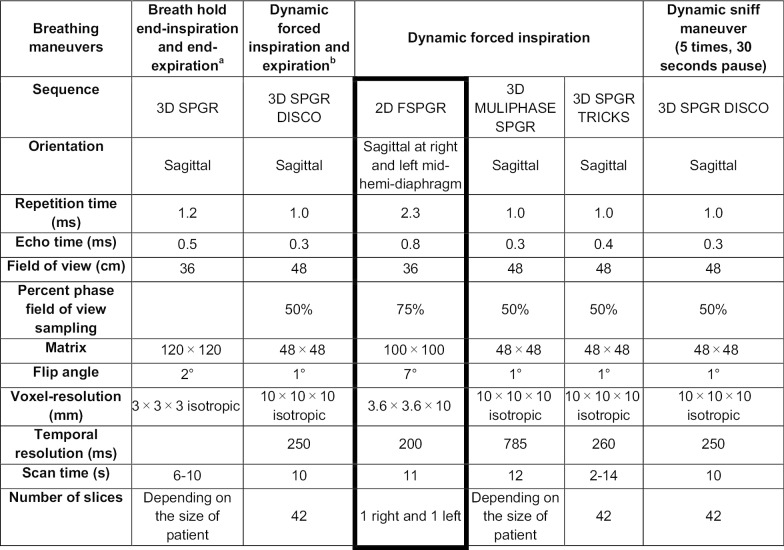
MRI protocol

MRI protocol and parameters on 3 T GE Signa 750 MRI (General Electric Healthcare, Milwaukee, USA) with a 32-channel torso coil. The acquisition for this part of the study is marked with thick borders and included 2D dynamic inspiratory SPGR multiphase sagittal acquisition at the level of the right and left mid-hemi-diaphragm. All dynamic inspiration acquisitions started with breath holding at end-expiration and ended with breath holding at end-inspiration. Dynamic forced expiration started with breath holding at end-inspiration and ended with breath holding at end-expiration

2D = bi-dimensional, 3D = three-dimensional, Disco = DIfferential Sub-sampling with Cartesian Ordering, SPGR = spoiled gradient echo, Tricks = time-resolved imaging of contrast kinetics, FSGPR = fast spoiled gradient echo

^a^After respective 3D SPGR inspiratory and expiratory localizer sequences

^b^Sequence will be repeated after 2D FSPGR sequence

In this study, we used dynamic sagittal bi-dimensional (2D) images, starting at breath holding at end-expiration, followed by a forced inspiration maneuver, and ending at breath holding at end-inspiration. The sagittal levels were manually selected at the right and left mid-hemidiaphragm using the three-dimensional (3D) breath holding acquisition at end-inspiration.

### Image segmentation

**Fig. 1:**
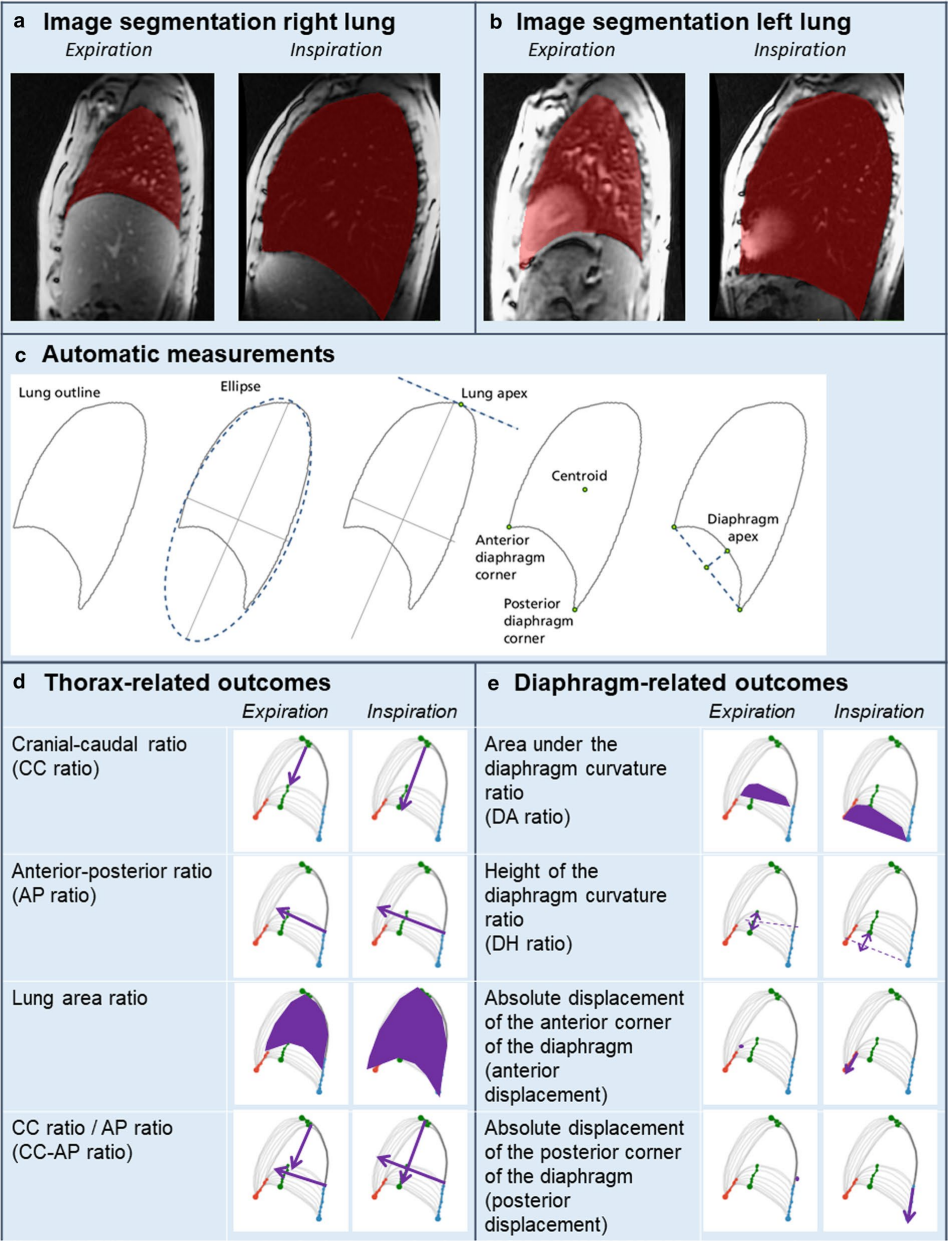
MRI measurements and outcomes. **a**, **b** Examples of manual image segmentation in the right and left lung at expiration and inspiration in an healthy control. **c** Steps of the automatic algorithm to define reference points to calculate the different outcome measures. **d** and **e** Thorax- and diaphragm-related outcomes. The grey lines depict the outlines of the lung in consecutive images from end expiration to end-inspiration. The red point indicates the anterior corner of the diaphragm and the blue point the posterior corner of the diaphragm. The green points indicate displacement of the lung apex and diaphragm apex. Purple arrows and purple areas indicate the outcome measurements

We selected the images during the inspiration maneuver, comprising ± 15 of 60 images. Manual image segmentation was performed by two observers (LH and AP) using ITK-SNAP (Version 3.6.0 Apr 1, 2017, Copyright © 1998–2017, www.itksnap.org) [22]. This free, open-source software application enables to manually indicate the area of the lung on the selected images. Anterior and posterior points of the insertion of the diaphragm were chosen as reference points for basal lung contours. For the left lung, the heart was included in the segmented lung area to optimally delineate the contours of the diaphragm (Fig. 1a, b).

### Automatic measurements

The segmentations were analyzed with a custom-built Python script (Python 3.6.3, https://www.python.org/, ©2001–2019. Python Software Foundation; SciPy 1.1.0, https://www.scipy.org/, ©2003–2019 SciPy developers) (Fig. 1c). The algorithm first determines the general orientation of the lung, to correct for minor differences in position between patients, by fitting an ellipse to the points on the lung outline. The lung apex is then defined as the highest point on the lung outline, measured in the direction of the major axis of the ellipse. The algorithm then detects the anterior and posterior costophrenic angles of the diaphragm, defined as the points with the largest distance to the centroid (center of gravity) in the lower part of the lungs. The diaphragm apex was defined as the point on the diaphragm contour that is furthest away from the linear line between both costophrenic angles of the diaphragm. Because for some patients this maximum-distance point was quite variable between consecutive frames, we used a derived diaphragm apex: therefore, the relative position of the diaphragm apex along the diaphragm contour was computed in each frame as a percentage of the diaphragm length; then, the median position of all frames was computed and this median relative distance was used for each frame to compute the derived diaphragm apex. All reference points were visual inspected by LH and GT to confirm that the points were correctly indicated.

### MRI outcome measures

Four thorax-related outcomes and four diaphragm-related outcomes were measured at end-expiration and end-inspiration (Fig. 1d, e). To correct for anatomical variations between individual patients, we calculated ratios by dividing end-inspiration outcomes by end-expiration outcomes.

### Thorax-related outcomes

The cranial-caudal (CC) distance was measured as the distance between the lung apex and diaphragm apex to evaluate diaphragmatic motion. The anterior–posterior (AP) distance is the longest distance from the front to the back of the lung to evaluate motion of the thoracic wall. To calculate the diaphragm motion relative to the motion of the thoracic wall, the CC-AP ratio was calculated by dividing the CC ratio by the AP ratio. The lung area is the area of the segmentation to evaluate the result of all respiratory muscles together. For the left hemi-diaphragm, lung area included the area of the heart to reduce the effect of heart size.

### Diaphragm-related outcomes

To evaluate the shape of the diaphragm, the area below the diaphragm curvature (DA) and the height of the diaphragm curvature (DH) were measured to calculate the DA ratio and DH ratio between inspiration and expiration. The DA is the area between the diaphragm contour and the linear line connecting both diaphragm corners. The DH is the perpendicular distance between the diaphragm apex and the linear line. In addition, the absolute displacements of the anterior and posterior costophrenic angles of the diaphragm were measured in consecutive images from end-expiration to end-inspiration.

### Validation of measurements

To validate automatic measurements, manual measurements of thorax related outcomes were performed by two independent observers (LH and AP). CC distance and AP distance were measured using ITK-SNAP and lung area was measured with Aw Server (2.0 General Electric Healthcare © 2009–2012). All automatically measured thorax-related outcomes were compared to manually measured outcomes. Second, both observers performed all measurements twice in 5 patients (9%) to calculate intra-observer variability and in 10 similar patients (19%) to calculate inter-observer variability. These measurements included new segmentations on end-expiration and end-inspiration images. We used intra-class correlation coefficients (ICC) based on absolute-agreement and two-way mixed-effects model [23]. Bland–Altman plots were inspected to assess possible systematic errors.

#### Statistical analysis

To investigate early signs of diaphragmatic weakness, we subdivided the Pompe patients into two subgroups: patients with normal spirometry results (FVC supine ≥ 80% than predicted and z-score ≥ −1.64) and patients with decreased spirometry results (FVC supine < 80% and z-score < −1.64).

To analyze differences in patient characteristics and pulmonary function outcomes between the two subgroups of Pompe patients and the healthy controls, we used the chi-square test for categorical variables and the Kruskal Wallis test for continuous variables. The Mann–Whitney test was used to test for differences in disease duration and duration of ERT between the two subgroups of Pompe patients. Overall differences in MRI outcomes and differences between healthy controls and the two subgroups of Pompe patients were analyzed using multiple linear regression analysis, with adjustment for significant differences in patient characteristics between the three subgroups. Residual plots were inspected to check the linearity assumption. The Spearman correlation coefficient was used to calculate the strength of association between the pulmonary function test outcomes and the MRI outcomes.

All analyses were performed on the left and right sides of the thorax. Because segmentation of the left hemidiaphragm is more cumbersome due to the heart, we report only the results of the right hemidiaphragm.

Statistical analysis was performed with SPSS for Windows (version 25, SPSS Inc, Chicago, IL). The significance level was set at *p* ≤ 0.05. Unless indicated otherwise, we report overall differences between the three subgroups. Differences between subgroups were corrected for multiple testing using Bonferroni’s method, resulting in a significance level of *p* ≤ 0.017. Due to the explorative character of the study, we did not adjust for multiple testing when reporting the different MRI outcomes.

## Results

### Participants characteristics and pulmonary function tests

We included 35 patients with Pompe disease (age 15–70 years) and 18 sex and age-matched healthy controls (Table 2). Thirteen patients had spirometry results in the normal range (FVC supine ≥ 80%); none of these patients had significant dyspnea or signs of nocturnal hypoventilation. Twenty-two patients had decreased spirometry results (FVC supine < 80%), one of whom used nocturnal non-invasive ventilation due to hypercapnia. Patients with normal spirometry results were younger, had a shorter disease duration since symptom onset, and fewer of them had been treated with ERT than patients with decreased spirometry results. Healthy controls had better FVC, MIP and MEP outcomes than Pompe patients. None of the patients had a significant kyphoscoliosis needing surgery or referral to an orthopedic surgeon.

**Table 2:** Participant characteristics

	Pompe patients with decreased spirometry results (FVC supine < 80%) (n = 22)	Pompe patients with normal spirometry results (FVC supine ≥ 80%) (n = 13)	Healthy controls (n = 18)	*p* value
Sex, number of males (%)	11 (50%)	7 (54%)	8 (44%)	0.869
Age, years	45 ± 16	31 ± 14	43 ± 14	0.030
Height, cm	178 ± 11	174 ± 9	178 ± 12	0.761
Weight, kg	76 ± 16	70 ± 13	79 ± 14	0.291
BMI, kg/m^2^	24 ± 4	23 ± 4	25 ± 3	0.262
Disease duration, years	16 ± 8	9 ± 9	–	0.018
Patients on ERT, n (%)	18 (82%)	6 (46%)	–	0.028
Duration of ERT, years	6 ± 5	6 ± 4	–	0.811
Non-invasive ventilation, n (%)	1 (3%)	0 (0%)	–	0.435
FVC upright, % of predicted	81 ± 11	97 ± 9	106 ± 8	< 0.001
FVC supine, % of predicted	60 ± 12	94 ± 9	102 ± 8	< 0.001
Δ FVC, % of predicted	21 ± 10	4 ± 4	4 ± 4	< 0.001
MIP, % of predicted	72 ± 23	88 ± 28	106 ± 25	0.001
MEP, % of predicted	80 ± 28	81 ± 28	112 ± 28	0.002

Characteristics and outcomes of pulmonary function tests of Pompe patients and healthy controls. Continues values are presented as mean ± standard deviation and were tested using the Mann Whitney or Kruskal Wallis tests. Categorical values are presented as number with percentage and were tested with the chi square test. P-values indicate overall differences between groups

BMI = body mass index, ERT = enzyme replacement therapy, FVC = forced vital capacity, Δ FVC = FVC upright – FVC supine, MIP = maximum inspiratory pressure, MEP = maximum expiratory pressure

### Thorax-related outcomes

**Fig. 2:**
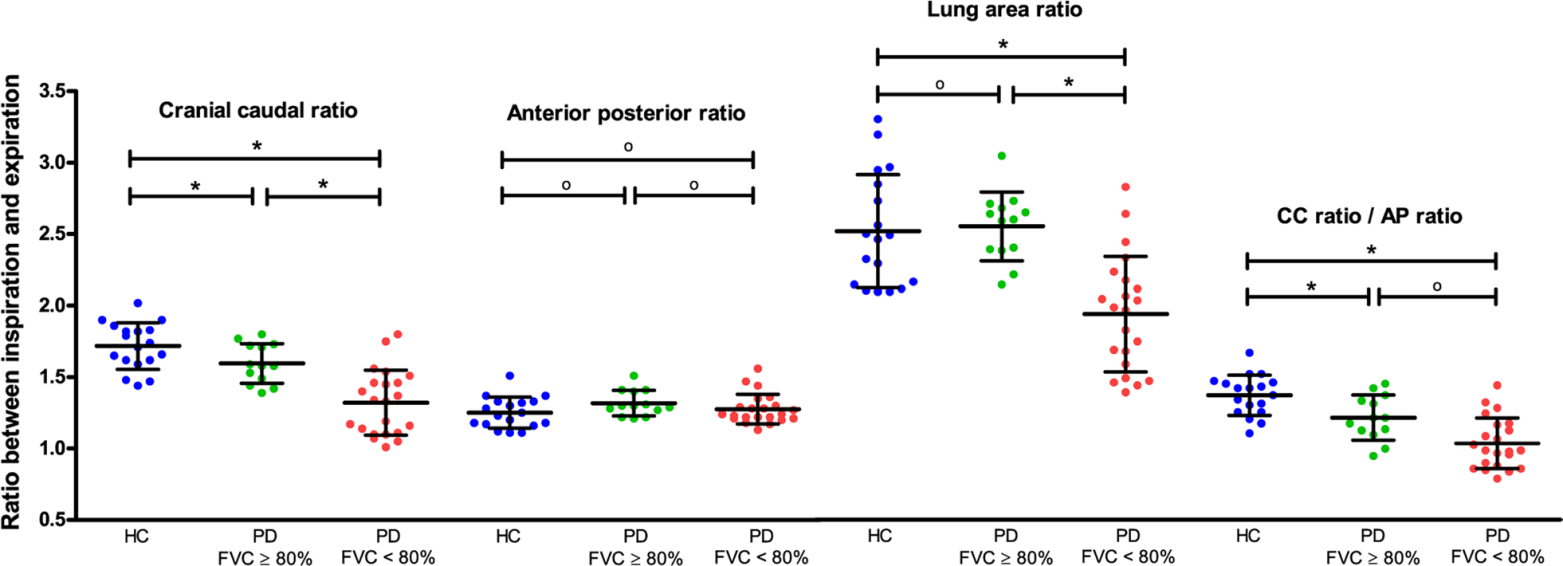
Thorax-related outcomes on the right side. The scatter plots present the thorax-related outcomes on the right side of the thorax in the two subgroups of Pompe patients and in healthy controls. Error bars show means with standard deviations. FVC = forced vital capacity in supine position. HC = healthy controls, PD = patients with Pompe disease. P-values < 0.017 are indicated with * as statistically significant, according to Bonferroni’s method for correcting for multiple testing. ^o^ means not statistically significant

The mean CC ratios were as follows: 1.72 in healthy controls, 1.60 in Pompe patients with normal spirometry results, and 1.32 in Pompe patients with decreased spirometry results (*p* < 0.001). The mean AP ratio did not differ significantly between the three subgroups. The mean lung area ratios were 2.54 in healthy controls, 2.57 in Pompe patients with normal spirometry results, and 1.95 in Pompe patients with decreased spirometry results (*p* = 0.001). The mean CC-AP ratios were 1.38 in healthy controls, 1.22 in Pompe patients with normal spirometry results, and 1.04 in Pompe patients with decreased spirometry results (*p* < 0.001). While patients with normal spirometry results had lower CC ratios and CC-AP ratios than healthy controls (*p* = 0.002 and *p* = 0.005), their lung area ratios were not significantly different (Fig. 2).

The agreement between automatic and manual measurements was excellent (ICC range 0.93–0.99), and the intra- and inter-observer agreement between manual measurements was good to excellent (ICC range 0.80–0.99).

### Diaphragm-related outcomes

**Fig. 3:**
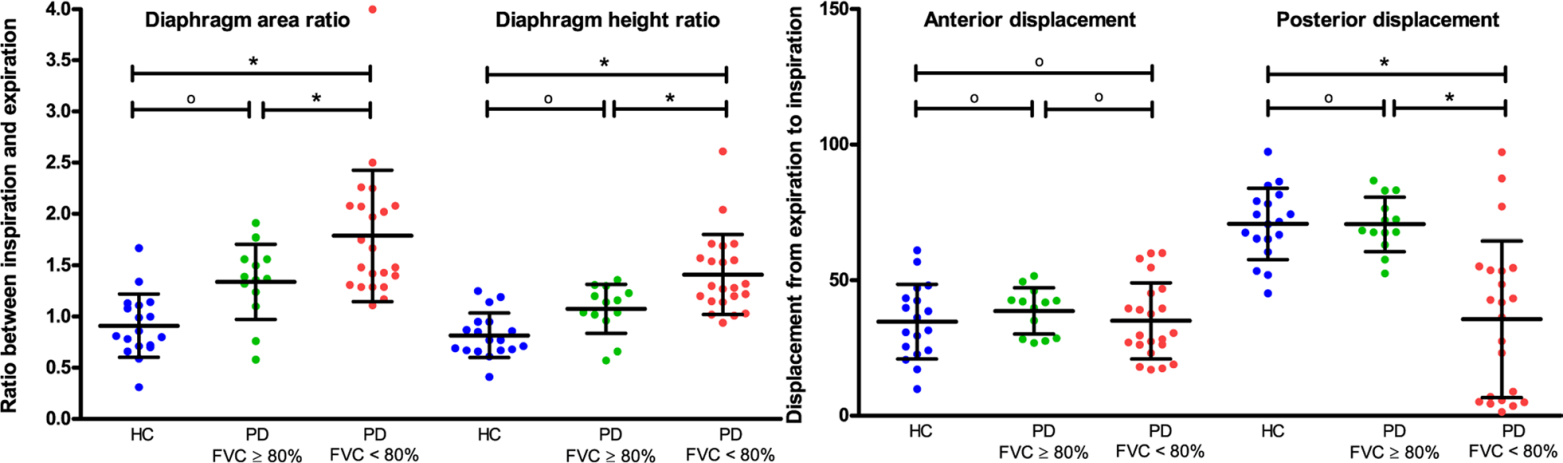
Diaphragm-related outcomes on the right side. The scatter plots present the diaphragm-related outcomes on the right side of the thorax in the two subgroups of Pompe patients and in healthy controls. Error bars show means with standard deviations. FVC = forced vital capacity in supine position. HC = healthy controls, PD = patients with Pompe disease. P-values < 0.017 are indicated with * as statistically significant, according to Bonferroni’s method for correcting for multiple testing. ^o^ means not statistically significant

During inspiration, DA and DH in healthy controls decreased, indicating a decreased curvature of the diaphragm. In Pompe patients, however, the curvature of the diaphragm remained equal or increased during inspiration. In healthy controls mean DA ratio was 0.91 and mean DH ratio 0.82, in Pompe patients with normal spirometry results mean DA ratio was 1.34 and mean DH ratio was 1.08, and in those with decreased spirometry results was mean DA ratio was 1.79 (*p* < 0.01) and the mean DH ratio was 1.41 (*p* < 0.01). In healthy controls, the absolute displacement of the posterior costophrenic angle of the diaphragm was larger than the displacement of the anterior costophrenic angle. Mean absolute displacement of the posterior costophrenic angle was 70.7 mm in healthy controls, 70.6 mm in Pompe patients with normal spirometry results, and 35.6 mm in Pompe patients with decreased spirometry results (*p* < 0.001). Mean absolute displacement of the anterior costophrenic angle did not differ between healthy controls and the two groups of Pompe patients. In patients with normal spirometry results, the DA and DH ratios were higher than in healthy controls (*p* = 0.088 and *p* = 0.034, which was not significant after Bonferroni’s correction) (Fig. 3).

### Levels of diaphragmatic weakness

**Fig. 4:**
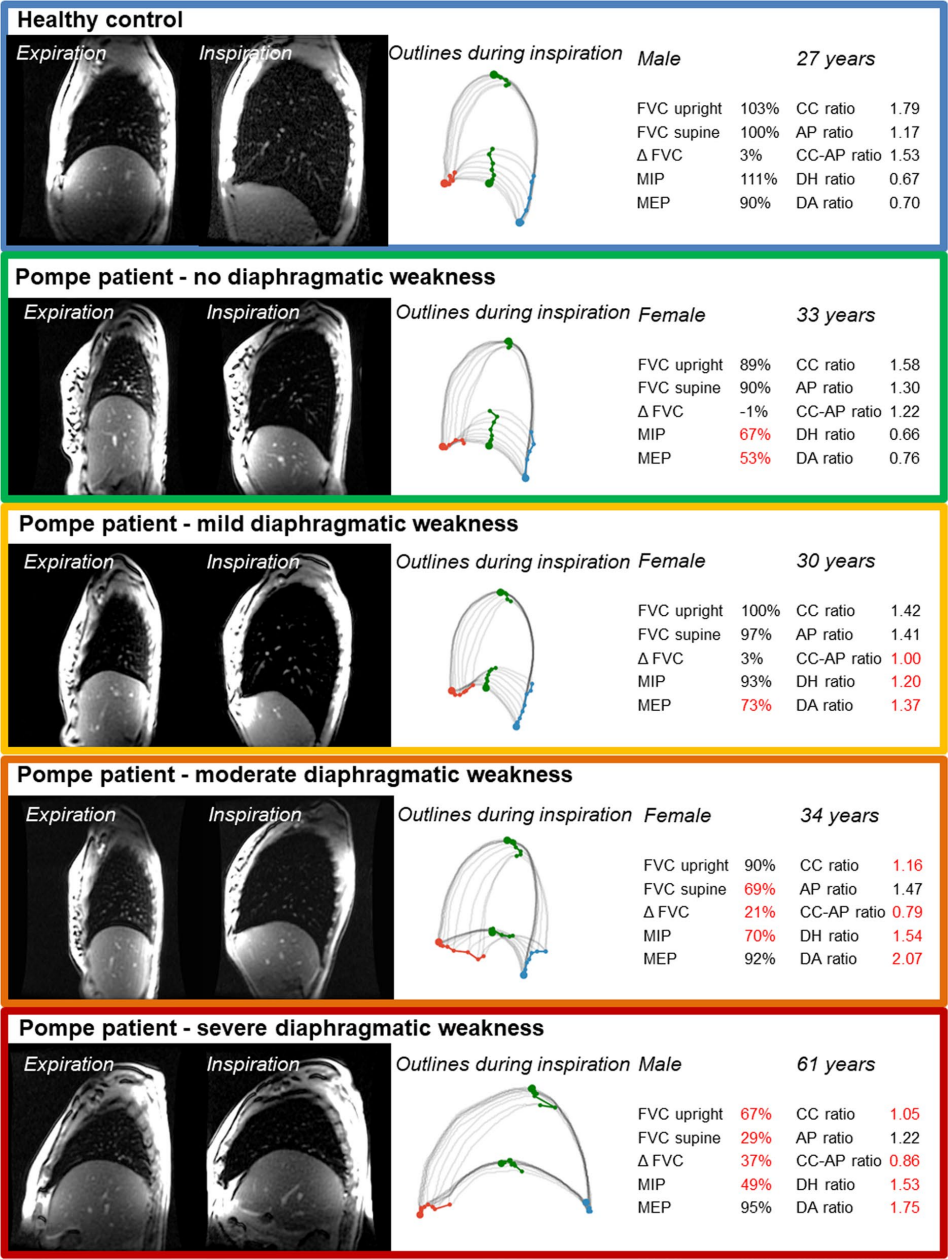
Levels of diaphragmatic weakness. Examples of a healthy control and of four Pompe patients with different levels of diaphragmatic weakness. The left-hand images show the sagittal MRI at end-expiration and end-inspiration. The grey outlines show the contours of the lungs from expiration to inspiration (bold lines). The blue point is the posterior corner of the diaphragm and the red point the anterior corner. The green points show the lung apex and diaphragm apex. The individual results of pulmonary function tests (% of predicted outcome) and MRI outcome ratios are also reported, with red numbers indicating abnormal outcomes compared to healthy controls. FVC = forced vital capacity, Δ FVC = FVC upright – FVC supine, MIP = maximum inspiration pressure, MEP = maximum expiration pressure, CC ratio = cranial-caudal ratio, AP ratio = anterior posterior ratio, CC-AP ratio = CC ratio / AP ratio, DH ratio = diaphragm height ratio, DA ratio = diaphragm area ratio

Using MRI to assess diaphragmatic weakness, different levels of severity could be indicted (Fig. 4). In patients with severe diaphragmatic weakness, the diaphragm shows hardly any motion and the CC ratio is ~ 1. Those with moderate diaphragmatic weakness still have diaphragmatic motion (CC ratio > 1), although it is lower than that in healthy controls. In patients with mild diaphragmatic weakness, the CC ratio is normal, but DA ratio and DH ratio are increased, indicating an increased curvature of the diaphragm compared to in healthy controls and patients without diaphragmatic weakness. Importantly, in patients with mild or moderate diaphragmatic weakness, AP ratio is still normal or increased, indicating an increased motion of the thoracic wall. The large contribution to inspiration of the thoracic wall relative to that of the diaphragm is also reflected by a lower CC-AP ratio.

### Correlation between pulmonary function tests and MRI-outcomes

CC ratio, CC-AP ratio and absolute displacement of the posterior corner of the diaphragm, outcomes evaluating the motion of the diaphragm, showed the best correlations with FVC supine (> 0.64) and Δ FVC (<−0.59) (Table 3). The correlations of these MRI outcomes with FVC upright and MIP, which lay between 0.32 and 0.52, showed that upright pulmonary function tests provided less information on the motion of the diaphragm than supine pulmonary function tests. The MRI outcomes that measure the curvature of the diaphragm during inspiration—the DA and DH ratios—showed moderate correlations with FVC supine (<−0.58) and Δ FVC (>0.48). The low correlations of MEP with all MRI outcomes (<0.3) indicate that this test does not have the sensitivity necessary for measuring diaphragmatic motion or shape.

**Table 3:** Correlation between MRI outcomes and pulmonary function tests outcomes

	FVC upright	FVC supine	Δ FVC	MIP	MEP
*Thorax-related outcomes*					
Cranial-caudal ratio	0.518***	0.680***	−0.654***	0.435**	0.187
Anterior–posterior ratio	0.035	0.097	−0.112	−0.008	0.040
Lung area ratio	0.473***	0.636***	−0.610***	0.398**	0.151
CC-AP ratio	0.509***	0.643***	−0.608***	0.403**	0.161
*Diaphragm-related outcomes*					
Diaphragm area ratio	−0.556***	−0.588***	0.484***	−0.355**	−0.293*
Diaphragm height ratio	−0.538***	−0.608***	0.552***	−0.382**	−0.270
Anterior displacement	0.089	0.201	−0.234	−0.065	−0.042
Posterior displacement	0.486***	0.650***	−0.598***	0.322*	0.021

Spearman correlation coefficients between thorax-related outcomes and diaphragm-related outcomes (ratios between end-inspiration and end-expiration outcomes) and outcomes of pulmonary function tests (% predicted). Significant correlations are indicated with *p < 0.05, **p < 0.01 or ***p < 0.001

CC-AP ratio = cranial caudal ratio / anterior posterior ratio. FVC = forced vital capacity, Δ FVC = FVC upright—FVC supine, MEP = mean expiratory pressure, MIP = mean inspiratory pressure

## Discussion

In this cross-sectional study we used 2D sagittal dynamic MRI to evaluate the motion and shape of the diaphragm in children and adults with non-classic Pompe disease who had normal or decreased spirometry results. We found that motion of the diaphragm is decreased in patients with Pompe disease, and that the curvature of the diaphragm in these patients is increased during inspiration. Interestingly, these MRI signs were observed even in Pompe patients whose spirometry results were still within the normal range. This means that MRI enables to detect early signs of diaphragmatic weakness. These results are important for Pompe patients, but potentially also for patients with other neuromuscular disease having respiratory dysfunction, because early recognition of impaired diaphragmatic function enables to start early interventions to prevent irreversible damage to the diaphragm.

Our observations show a clear dissociation between the loss of function of the diaphragm and the intercostal musculature. Diaphragmatic weakness is common in adults with Pompe disease, and earlier 2D and 3D MRI pilot studies in small groups of Pompe patients with decreased spirometry results also found decreased motion of the diaphragm [15–18]. Interestingly, this is different compared to studies in patients with Duchenne muscular dystrophy, in whom the motion of the chest wall as well as the diaphragm are reduced [24]. As we wished to investigate whether early signs of diaphragmatic weakness could also be observed in Pompe patients with normal spirometry results, we conducted the current MRI study. A remarkable finding was that while motion of the thoracic wall still lay in the normal range, motion of the diaphragm was already less than in healthy controls. Early involvement of the diaphragm might be an explanation for the presentation with respiratory symptoms without limb girdle muscle weakness in in a subset of patients with Pompe disease [25, 26].

A second finding was that Pompe patients had an increased curvature of the diaphragm during inspiration compared to healthy controls. We hypothesize that the aberrant shape of the diaphragm during inspiration is caused by diaphragmatic weakness. In healthy controls, during inspiration, intrathoracic pressure is reduced and abdominal pressure is increased by the downward movement of the diaphragm and the outward movement of the chest wall [27, 28]. In patients with severe diaphragmatic weakness, however, the lower intrathoracic pressure and impaired tonus of the diaphragm result in an upward paradoxical displacement of the diaphragm [28]. As many Pompe patients also have abdominal muscle weakness, abdominal pressure will not increase sufficiently during inspiration. Consequently, there is little or no paradoxical movement of the diaphragm, but only a paradoxical increased curvature of the diaphragm during inspiration.

An important question is why the preferential weakness of the diaphragm compared to the intercostal muscles occurs. In healthy adults, the diaphragm comprises an equal distribution of slow-twitch type-1 fibers and fast-twitch type-2 fibers, while the intercostal muscles contain a higher proportion of fast-twitch type-2 fibers [27, 29, 30]. In Pompe patients, it is known that damage to skeletal muscle includes both muscle fiber types, but there have been no studies on the involvement of specific respiratory-muscle fiber types [31]. Early damage of the diaphragm however could also be facilitated by the fact that this muscle and tendon plate is relatively thin [32].

As our study showed that FVC supine and the difference between FVC upright and FVC supine (Δ FVC) had the highest correlations with the motion of the diaphragm as measured with MRI, these pulmonary function tests are important parameters to diagnose diaphragmatic weakness. While weakness of the diaphragm is usually clear in patients with FVC supine < 80%, our dynamic MRI protocol can be used to demonstrate a different shape or reduced motion of the diaphragm in patients with an FVC ≥ 80%, which is considered to be in the normal range [11].

Ultrasound is another technique to investigate the diaphragm. In Pompe patients, it showed a reduced diaphragmatic thickness and motion and a good correlation with FVC in seated and supine position [33]. While ultrasound logistically might be more easy to perform, a comparison between diaphragmatic motion and motion of intercostal muscles and a more detailed evaluation of the curvature of the diaphragm is not possible. Therefore, detection of slight diaphragmatic weakness in an early stage of Pompe disease using ultrasound will likely be difficult.

By using our new 2D sagittal dynamic MRI protocol, we were able to identify reliable automatically measured outcome measures to characterize the motion and shape of the diaphragm. Our automatic measurements are operator independent and showed excellent correlation with manual measurements.

Our study however has some limitations. Spirometry-controlled MRI is logistically more difficult and expensive than regular pulmonary function tests. Second, segmentations were performed manually and were time consuming. Further development of automatic measurements, including automatic segmentations would be useful. Additional investigation is required to determine whether this chest MRI technique may also be useful to detect a potential neuromuscular cause in patients who present with dyspnea and/or respiratory failure of unknown origin. To generate reference values for our MRI outcomes, the population of healthy controls should be further increased.

## Conclusions

We have demonstrated that MRI is a sensitive tool for detecting early stages of diaphragmatic weakness in patients with Pompe disease, even when spirometry results are within the normal range. MRI provides insight into the process of increasing diaphragmatic weakness, and when FVC starts to decline, possible irreversible functional changes of the diaphragm may have already taken place. This may explain why respiratory function as assessed by spirometry responds relatively poorly to ERT. Potentially, the MRI outcome measures may be helpful to optimize the start of treatment, and to assess diaphragmatic function and therapy response over time, not only in Pompe disease, but also in other neuromuscular diseases associated with respiratory dysfunction.

## Data Availability

The datasets containing anonymized MRI outcomes during and/or analysed during the current study, anonymized image segmentations and the algorithm to analyze segmentations are available from the corresponding author on reasonable request. In order to protect privacy and confidentiality of the study participants, complete MRI data is not publicly available.

## Abbreviations

2D: Bi-dimensional
3D: Three-dimensional
AP: Anterior–posterior
CC: Cranial-caudal
DA: Diaphragm area
DH: Diaphragm height
ERT: Enzyme Replacement Therapy
FVC: Forced Vital Capacity
ICC: Intra-class Correlation Coefficients
MEP: Maximum Expiratory Pressure
MIP: Maximum Inspiratory Pressure
